# Preclinical testing of dabigatran in trypsin-dependent pancreatitis

**DOI:** 10.1172/jci.insight.161145

**Published:** 2022-11-08

**Authors:** Zsófia Gabriella Pesei, Zsanett Jancsó, Alexandra Demcsák, Balázs Csaba Németh, Sandor Vajda, Miklós Sahin-Tóth

**Affiliations:** 1 Department of Surgery, University of California Los Angeles, Los Angeles, California, USA.; 2 Department of Biomedical Engineering, Boston University, Boston, Massachusetts, USA.

**Keywords:** Inflammation, Therapeutics, Genetic diseases, Mouse models, Thrombin

## Abstract

Pancreatitis, the inflammatory disorder of the pancreas, has no specific therapy. Genetic, biochemical, and animal model studies revealed that trypsin plays a central role in the onset and progression of pancreatitis. Here, we performed biochemical and preclinical mouse experiments to offer proof of concept that orally administered dabigatran etexilate can inhibit pancreatic trypsins and shows therapeutic efficacy in trypsin-dependent pancreatitis. We found that dabigatran competitively inhibited all human and mouse trypsin isoforms (*K_i_* range 10–79 nM) and dabigatran plasma concentrations in mice given oral dabigatran etexilate well exceeded the *K_i_* of trypsin inhibition. In the *T7K24R* trypsinogen mutant mouse model, a single oral gavage of dabigatran etexilate was effective against cerulein-induced progressive pancreatitis, with a high degree of histological normalization. In contrast, spontaneous pancreatitis in *T7D23A* mice, which carry a more aggressive trypsinogen mutation, was not ameliorated by dabigatran etexilate, given either as daily gavages or by mixing it with solid chow. Taken together, our observations showed that benzamidine derivatives such as dabigatran are potent trypsin inhibitors and show therapeutic activity against trypsin-dependent pancreatitis in *T7K24R* mice. Lack of efficacy in *T7D23A* mice is probably related to the more severe pathology and insufficient drug concentrations in the pancreas.

## Introduction

There is no specific pharmacological therapy for the inflammatory disorders of the exocrine pancreas, which include acute pancreatitis (AP), recurrent acute pancreatitis (RAP), and chronic pancreatitis (CP) (1). Pancreatitis often presents as a progressive, relapsing-recurring disease, in which a sentinel episode of AP progresses to RAP, and eventually to CP, driven by underlying genetic and environmental risk factors (1, 2). Studies on genetic risk factors associated with the AP-RAP-CP sequence (denoted as CP from here on out) revealed that ectopic, intrapancreatic activation of the digestive protease precursor trypsinogen to its active form trypsin is responsible for disease onset and progression (3–5). Genetic alterations increase intrapancreatic trypsin activity by accelerating trypsinogen autoactivation, and/or by diminishing protective mechanisms, such as trypsinogen degradation by chymotrypsin C and trypsin inhibition by the serine protease inhibitor Kazal type 1. This disease model has been designated as the trypsin-dependent pathway of genetic risk in CP, which not only formulates a mechanistic framework for CP development but also identifies a clear therapeutic target, i.e., trypsin (4, 5).

Considering the long history of biochemical investigations into the function of trypsin, and the availability of a large number of natural and synthetic trypsin inhibitors, it seems surprising that no effective antitrypsin therapy has been developed so far to treat or prevent pancreatitis. Although bovine pancreatic trypsin inhibitor (aprotinin) (6) and the *p*-guanidino-benzoate derivatives gabexate (FOY), camostat (FOY-305) (7), and nafamostat (FUT-175) (8) seemed effective in rodent experiments (9–16), their clinical efficacy has been either variable or disappointing (17–22 and references therein). This apparent contradiction can be largely explained by the fact that early preclinical experiments did not use mouse models of trypsin-dependent pancreatitis. Similarly, clinical trials did not focus on trypsin-dependent disease, which could be verified by appropriate genetic testing. Finally, the compounds evaluated had inhibitory activity against a range of proteases other than trypsin, which further confounds interpretation of the results from preclinical and clinical trials. Despite their dubious efficacy, nafamostat and gabexate infusions are approved to treat AP, and oral camostat is used for the management of pain associated with CP in Japan. More recently, the COVID-19 pandemic sparked revived awareness in these drugs, as camostat and nafamostat have been identified as strong inhibitors of the transmembrane serine protease 2, which is required for cellular entry of the SARS-CoV-2 virus (23, 24). Several clinical trials have been initiated to test efficacy against COVID-19, and the results may also inform future clinical trials in pancreatitis with respect to dosing and side effects (25–28).

**Figure 1:**
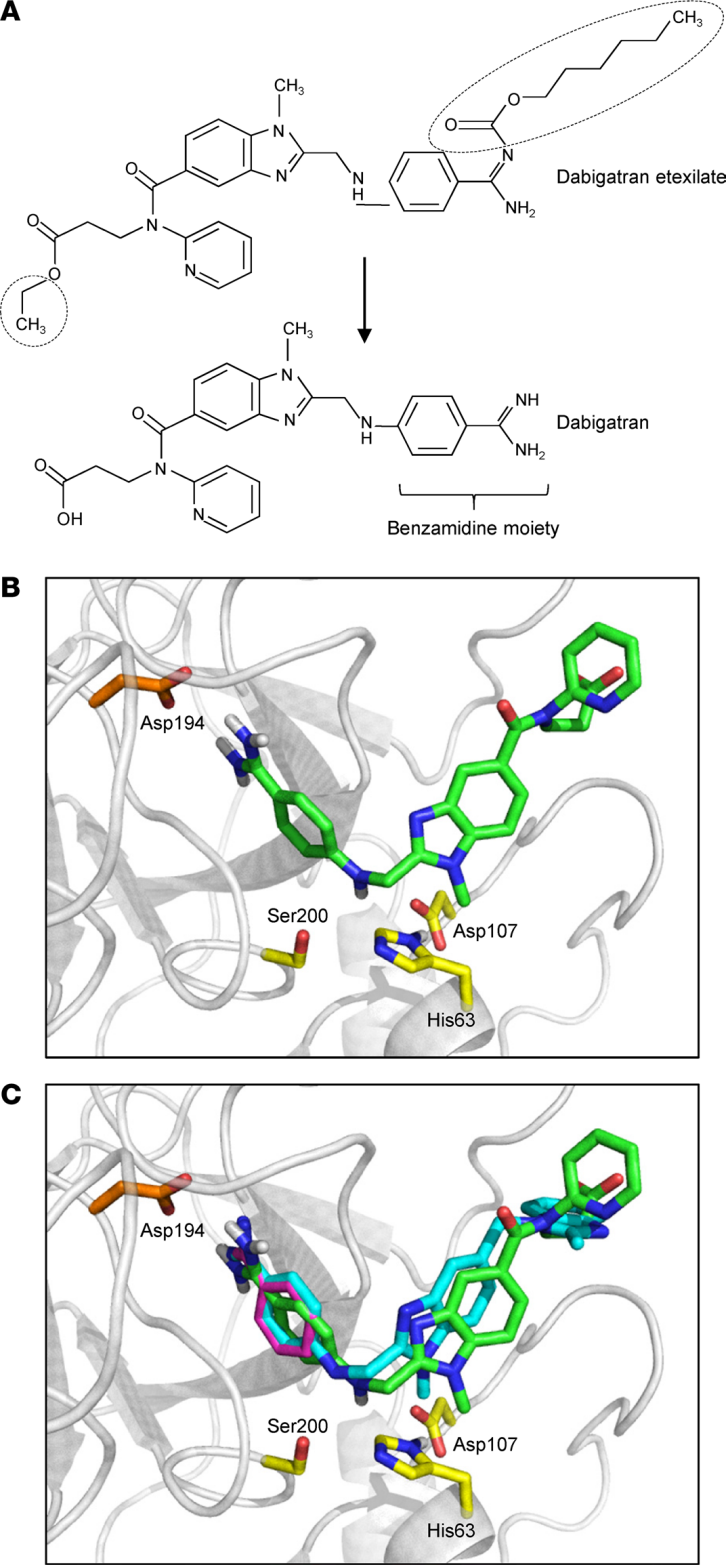
Modeling dabigatran binding to trypsin. (**A**) Chemical structure of dabigatran and its prodrug dabigatran etexilate. Differences are encircled. The benzamidine moiety of dabigatran is indicated. (**B**) Dabigatran (shown as green sticks) docked to human mesotrypsin (PDB structure 1H4W, shown as gray cartoon). Also indicated are the side chains of the catalytic triad His63, Asp107, and Ser200 (corresponding to His57, Asp102, and Ser195 in conventional crystallographic numbering) and Asp194 (Asp189) at the bottom of the specificity pocket (yellow and orange sticks, respectively). (**C**) Superimposition of trypsin-bound dabigatran, with benzamidine (from PDB structure 1H4W, magenta) and the benzamidine-derivative, dual-specificity thrombin and factor Xa inhibitor R11 cocrystallized with bovine trypsin (from PDB structure 1G36, cyan). The figures were created with the PyMOL Molecular Graphics System (https://pymol.org/2/).

In addition to the renewed interest in small–molecular weight trypsin inhibitors, novel mouse models of trypsin-dependent pancreatitis have been developed recently. Our laboratory generated several knockin mouse lines carrying human pancreatitis-associated mutations in the native mouse cationic trypsinogen (isoform T7) (29–32). Concurrently, the laboratory of Baoan Ji created transgenic models harboring wild-type or mutated forms of the serine protease 1 and 2 (*PRSS1* and *PRSS2*) genes that encode human cationic and anionic trypsinogens, respectively (33–36). All published mouse models develop either spontaneous, progressive pancreatitis driven by trypsinogen autoactivation or exhibit heightened sensitivity to cerulein-induced experimental pancreatitis. Remarkably, in their 2020 publication (34), the Ji group reported that the anticoagulant dabigatran etexilate markedly improved cerulein-induced pancreatitis in transgenic mice carrying human *PRSS1* with the p.R122H mutation (named PRSS1^R122H^ mice by the authors). Dabigatran etexilate (brand name Pradaxa) is routinely used worldwide as an orally active, reversible thrombin inhibitor for the anticoagulation of patients with venous thromboembolism or increased risk of stroke due to atrial fibrillation (37–39). It is a prodrug, which is absorbed from the gastrointestinal tract and becomes converted in the blood by nonspecific esterases to its active form, dabigatran (Figure 1A) (40). Dabigatran is a benzamidine derivative, and it has been reported to inhibit human thrombin reversibly with a *K_i_* of 4.5 nM and bovine trypsin with a *K_i_* of 50.3 nM (41). We hypothesized that the observed therapeutic effect of dabigatran in the PRSS1^R122H^ transgenic mice was due to trypsin inhibition in the pancreas rather than inhibition of thrombin in the circulation. To test this notion, here we compared the inhibitory activity of dabigatran against a panel of human and mouse trypsin isoforms in vitro and performed preclinical drug testing studies using 2 mouse models of trypsin-dependent pancreatitis. *T7D23A* and *T7K24R* mice carry the p.D23A and p.K24R mutations in mouse T7 trypsinogen, which are analogous to the human pancreatitis-associated *PRSS1* mutations p.D22G and p.K23R, respectively (29, 30). The p.D23A and p.K24R mutations increase autoactivation of mouse cationic trypsinogen by 50-fold and 5-fold, respectively. *T7D23A* mice are born with a normal pancreas but develop spontaneous, progressive pancreatitis at an early age (29). In contrast, *T7K24R* mice have no spontaneous phenotype, but they develop severe cerulein-induced AP followed by a progressive, CP-like disease (30, 31). These 2 models allowed us to test the effect of dabigatran etexilate under various conditions, i.e., cerulein-induced pancreatitis (*T7K24R*) versus spontaneous disease (*T7D23A*). Furthermore, we measured the plasma concentration of dabigatran after oral administration of dabigatran etexilate, in an attempt to correlate drug levels with trypsin-inhibitory activity and therapeutic efficacy. The results support and extend the potential utility of dabigatran and other benzamidine derivatives in pancreatitis therapy and highlight specific challenges the drug development process must overcome before advancement to clinical practice.

## Results

### Inhibition of trypsin by dabigatran.

We hypothesized that the reported efficacy of dabigatran etexilate in pancreatitis (34) is related to the trypsin-inhibitory activity of dabigatran. As a benzamidine derivative (Figure 1A), dabigatran is expected to inhibit trypsin-like enzymes competitively; however, this inhibitory activity against human and mouse trypsin isoforms has not been demonstrated before to our knowledge.

First, we used homology-based docking to demonstrate that dabigatran can bind to the specificity pocket of trypsin. In our model, showing dabigatran docked to human mesotrypsin (Protein Data Bank [PDB] 1H4W) (42), the amidine moiety of dabigatran interacts with the side chain of Asp194 at the bottom of the specificity pocket, and the *N*-methyl-benzimidazole scaffold that bridges the benzamidine and the distal pyridine ring and propanoic acid end is positioned above the catalytic triad (Figure 1B). The benzamidine moiety of dabigatran overlaps with the bound benzamidine of the 1H4W mesotrypsin structure (Figure 1C). The docked conformation of dabigatran is also similar to that of the benzamidine-derivative dual-specificity thrombin and factor Xa inhibitor R11 (PubChem SID 820345) cocrystallized with bovine trypsin (PDB structure 1G36) (43). The latter compound and dabigatran both have the benzamidine group deep in the specificity pocket, connected to an *N*-methyl-benzimidazole moiety. Dabigatran and R11 differ in the groups at the distal end. R11 has a second methyl-benzimidazole group that lies flat on the protein surface. In contrast, in the docked structure of dabigatran, the carboxyl group of the distal propanoic acid is partially solvent exposed, and the pyridine ring is perpendicular to the plain of the methyl-benzimidazole in R11. Most atoms of dabigatran, including the benzamidine group, are shifted by 0.6–0.8 Å toward the catalytic triad relative to the same atoms in R11 (Figure 1C).

**Figure 2:**
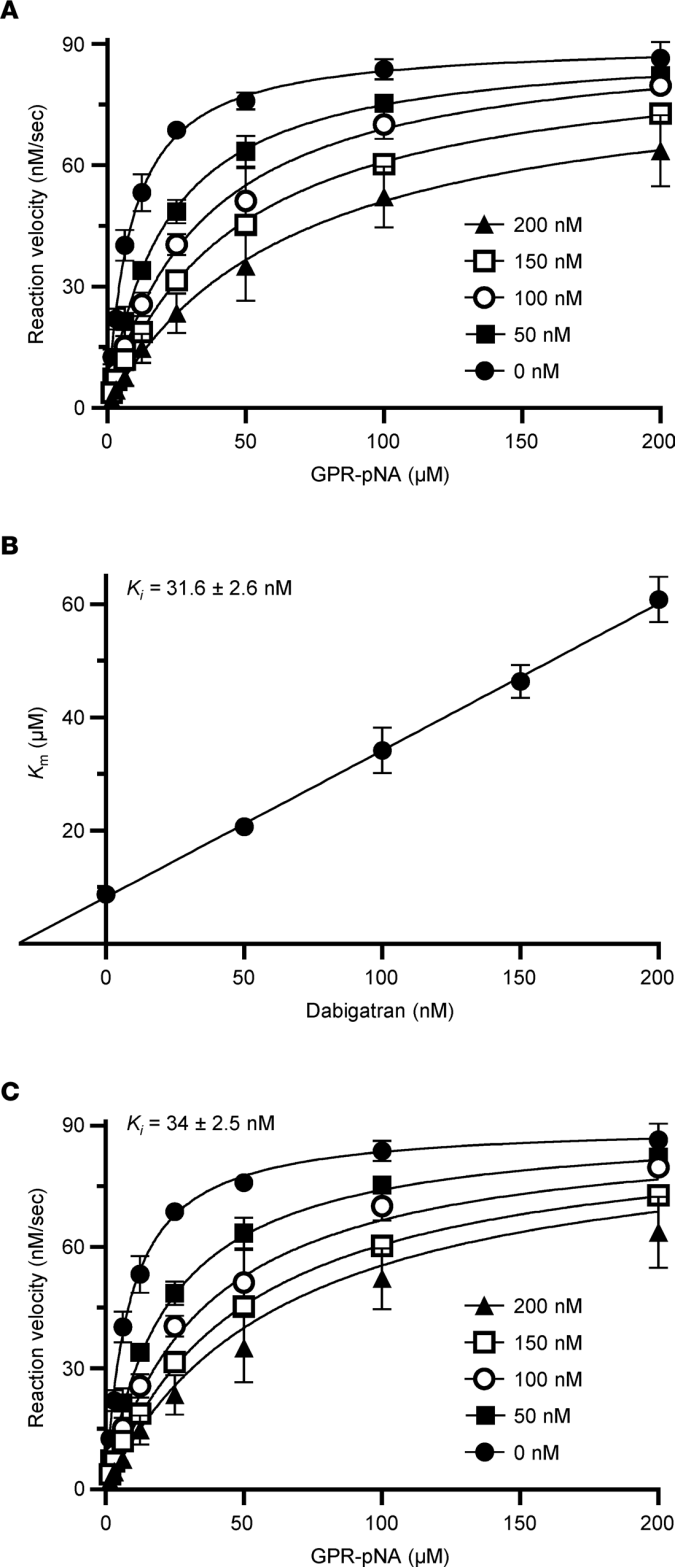
Inhibition of trypsin by dabigatran. Representative graphs of the kinetic assays using mouse anionic trypsin (isoform T8) are shown. Three experiments were performed. For clarity and convenience, the mean of the data points was plotted with standard deviation error bars, even though each experiment was analyzed separately. (**A**) Initial rate of trypsin activity as a function of substrate concentration in the absence and presence of increasing dabigatran concentrations. Rates were measured with 1 nM trypsin and the indicated concentrations of the *N*-CBZ-Gly-Pro-Arg-*p*-nitroanilide (GPR-pNA) trypsin substrate. Data sets for given dabigatran concentrations were *individually fitted* to the Michaelis-Menten equation. (**B**) Calculation of the competitive inhibitory constant (*K_i_*) of dabigatran (mean ± standard deviation, *n* = 3). The *K_m_* values derived from the saturation curves in **A** were plotted as a function of the dabigatran concentration. The *K_i_* was then determined by dividing the *y* axis intercept with the slope of the linear fit. This value corresponds to the negative of the *x* axis intercept. (**C**) Calculation of the *K_i_* of dabigatran by global fitting (mean ± standard deviation, *n* = 3). The data points from **A** were *globally fitted* to the competitive inhibition equation, as described in Methods.

Next, we performed enzymatic measurements to compare the effect of benzamidine and dabigatran against human trypsin isoforms PRSS1 (serine protease 1, cationic trypsin), PRSS2 (serine protease 2, anionic trypsin), and PRSS3 (serine protease 3, mesotrypsin) and mouse trypsin isoforms T7 (cationic trypsin), T8, T9, and T20 (anionic trypsins). The trypsin isoforms were produced recombinantly, but human PRSS1 and PRSS2 were also purified from pancreatic juice in their native form, which contains a sulfate group on Tyr154. As a universal reference molecule, commercial bovine cationic trypsin was also assayed. Figure 2A demonstrates a representative experiment for the determination of the competitive inhibitory constant (*K_i_*). As described in Methods, we calculated the *K_i_* values by either individually (Figure 2B) or globally (Figure 2C) fitting the substrate saturation curves and obtained comparable results. Table 1 and Table 2 indicate the *K_m_* and *k_cat_* values determined in the absence and presence of increasing benzamidine and dabigatran concentrations. We show these data to demonstrate that the *k_cat_* values remained constant within experimental error across all inhibitor concentrations tested, supporting the competitive nature of the inhibition. Table 3 lists the *K_i_* values for benzamidine and dabigatran. Benzamidine inhibited trypsin with micromolar *K_i_* values (range 3.3–20.6 μM and 4.2–22.6 μM by individual and global fit analysis, respectively), while dabigatran was an about 200- to 400-fold stronger inhibitor, exhibiting nanomolar *K_i_* values (range 10–65 nM and 10.3–78.9 nM by individual and global fit analysis, respectively). Anionic trypsin isoforms were inhibited slightly stronger by benzamidine than cationic trypsins; however, this trend was less conspicuous with dabigatran. Reassuringly, the *K_i_* values reported for dabigatran against bovine trypsin (41) and measured in our experiments were essentially identical. Dabigatran inhibited human trypsins as well as or slightly better than mouse trypsins, suggesting that results from preclinical mouse experiments should be relevant to human clinical trials. The experiments demonstrate that derivatives of benzamidine, such as dabigatran, can have highly improved inhibitory activity against trypsin and are universally effective against various trypsin paralogs.

### Plasma levels of dabigatran after oral administration of dabigatran etexilate.

**Figure 3:**
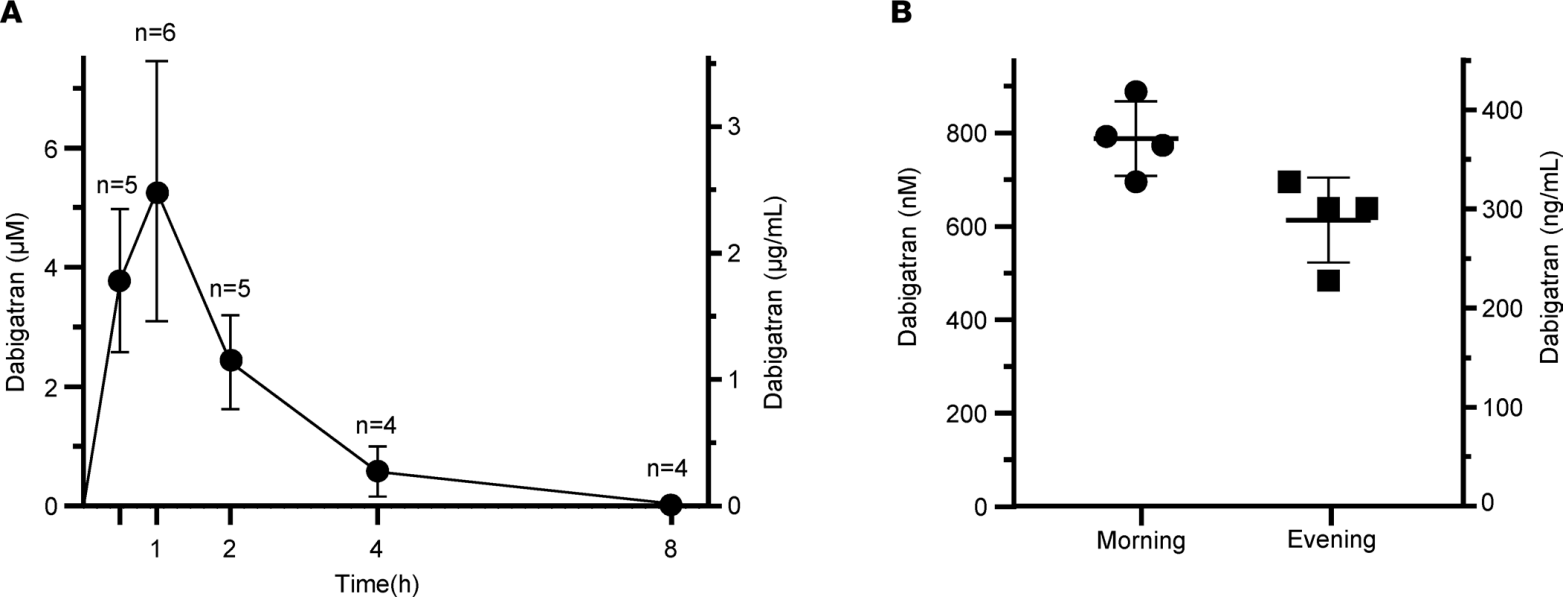
Dabigatran plasma concentrations in C57BL/6N mice following oral administration of dabigatran etexilate. Dabigatran levels were measured as described in Methods. (**A**) Mice (6–8 weeks of age) were given a single dose of 100 mg/kg dabigatran etexilate by oral gavage. Mice were euthanized at the indicated times. Data represent mean ± standard deviation (*n* = 4–6, as indicated). (**B**) Mice (12 weeks of age) were fed regular chow containing dabigatran etexilate at 10 mg/g concentration for 7 days. Mice were euthanized on day 8 either in the morning (9 am) or in the evening (5 pm). Mean dabigatran concentrations (± standard deviation, *n* = 4) measured at these time points were 787 ± 79 and 614 ± 91 nM, respectively.

Before embarking on experiments with trypsin-dependent pancreatitis models, we measured plasma concentrations of dabigatran in C57BL/6N mice after oral administration of the prodrug dabigatran etexilate. First, we performed intragastric gavage of a single dose (100 mg/kg) and followed plasma levels up to 8 hours. As shown in Figure 3A, dabigatran levels sharply rose to micromolar values within 30 minutes of oral gavage and peaked around 1 hour, after which time levels steadily decreased, with very little dabigatran measurable at the 4- and 8-hour time points. Importantly, peak concentrations of dabigatran were more than 2 orders of magnitude above the *K_i_* values measured for trypsin inhibition. Second, we fed mice with solid chow containing dabigatran etexilate (10 mg/g) for 1 week and measured their plasma dabigatran concentration. Compared with acute administration of the prodrug by gavage, chronic feeding resulted in lower but steadier plasma concentrations (Figure 3B), with most values falling in the 600–800 nM range. This drug level is still more than 10-fold higher than *K_i_* values of dabigatran against mouse trypsins.

### Effect of dabigatran etexilate on cerulein-induced pancreatitis in T7K24R mice.

**Figure 4:**
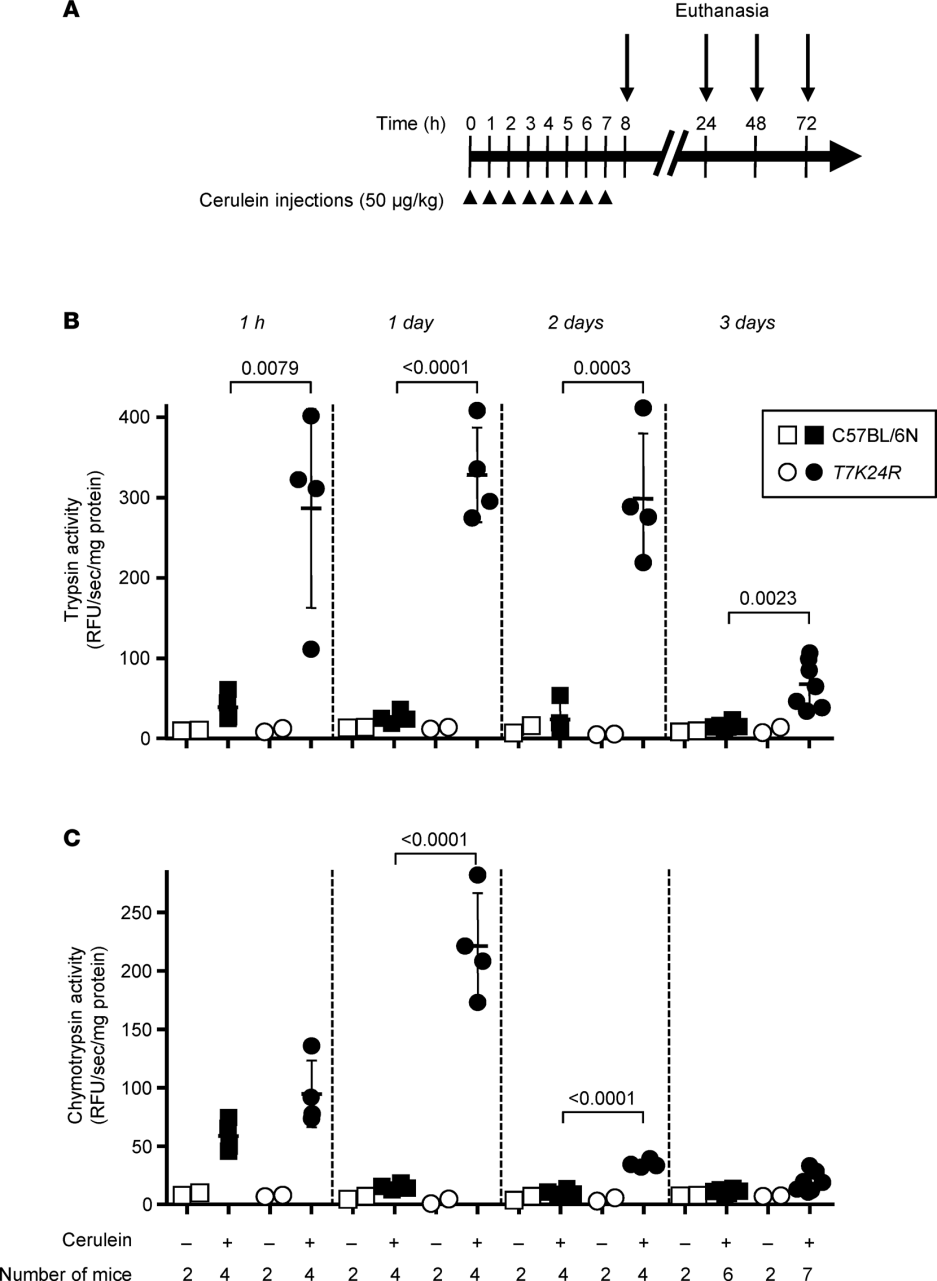
Intrapancreatic trypsin and chymotrypsin activity in *T7K24R* mice given saline or cerulein. (**A**) Experimental design. C57BL/6N and *T7K24R* mice (8–9 weeks of age) were treated with 8 hourly injections of saline or cerulein (arrowheads). Animals were euthanized at the indicated times (arrows), and the pancreas was freshly homogenized and assayed, as described in Methods. (**B**) Trypsin activity. (**C**) Chymotrypsin activity. Individual data points with mean and standard deviation are shown. The difference of means between the groups was analyzed by 1-way ANOVA with Tukey-Kramer post hoc test.

The *T7K24R* mouse strain carries the p.K24R mutation in mouse cationic trypsinogen (isoform T7), which is analogous to the p.K23R pancreatitis-associated human *PRSS1* mutation (30). The mutation increases autoactivation of trypsinogen about 5-fold and thereby sensitizes the pancreas to experimental pancreatitis. We recently demonstrated that cerulein-induced pancreatitis in *T7K24R* mice is progressive; and after the acute episode, marked acinar atrophy develops with fibrosis and macrophage infiltration (31). The acinar cell dropout is most prominent on days 4–6 and involves essentially the entire pancreas. This trypsin-dependent outcome is convenient to monitor and quantify. Before testing the effect of dabigatran etexilate, we characterized intrapancreatic trypsin and chymotrypsin activity in *T7K24R* mice after 8 hourly injections of saline or cerulein (Figure 4A). Protease activities were measured at 1 hour, 1 day, 2 days, and 3 days after the cerulein injections. Relative to cerulein-treated C57BL/6N mice, pancreatic trypsin activity in *T7K24R* mice was at least 10-fold elevated (Figure 4B), and this high value persisted on days 1 and 2, finally diminishing on day 3, as acinar atrophy develops (31). Pancreatic chymotrypsin activity was also significantly higher in *T7K24R* mice, with peak activity (20-fold higher than in C57BL/6N mice) seen on day 1, which sharply declined by days 2 and 3 (Figure 4C). The different temporal kinetics of intrapancreatic trypsin and chymotrypsin activities are intriguing, although an explanation is not readily apparent. As expected, no intrapancreatic protease activation was observed in saline-treated control mice. The high trypsin activity in the pancreas of cerulein-treated *T7K24R* mice suggests that trypsin-inhibitory therapy should be efficacious against pancreatitis in this model.

**Figure 5:**
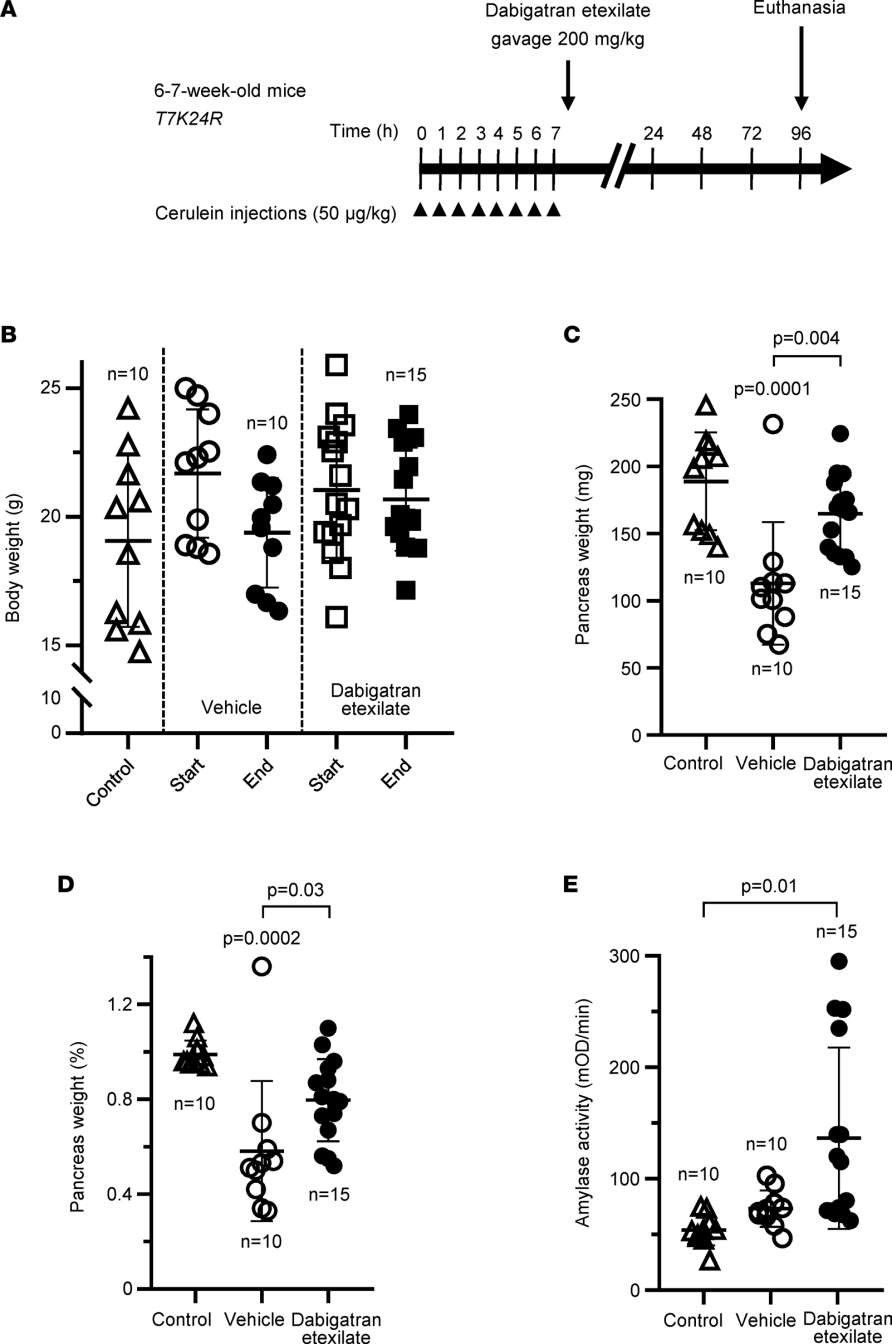
Effect of dabigatran etexilate on cerulein-induced pancreatitis in *T7K24R* mice. (**A**) Experimental design. Mice were given 8 hourly injections of cerulein (arrowheads), followed by a single oral gavage of 200 mg/kg dabigatran etexilate or vehicle administered 30 minutes after the last cerulein injection. Mice were euthanized 96 hours from the first cerulein injection. (**B**) Body weight at the start and end of the experiment. (**C**) Pancreas weight in milligrams. (**D**) Pancreas weight as percentage of body weight. (**E**) Plasma amylase activity (4 μL assayed). Control mice represent age-matched *T7K24R* mice without pancreatitis. Individual data points with mean and standard deviation are shown. The difference of means between the groups was analyzed by 1-way ANOVA with Tukey-Kramer post hoc test. mOD, milli–optical density.

**Figure 6:**
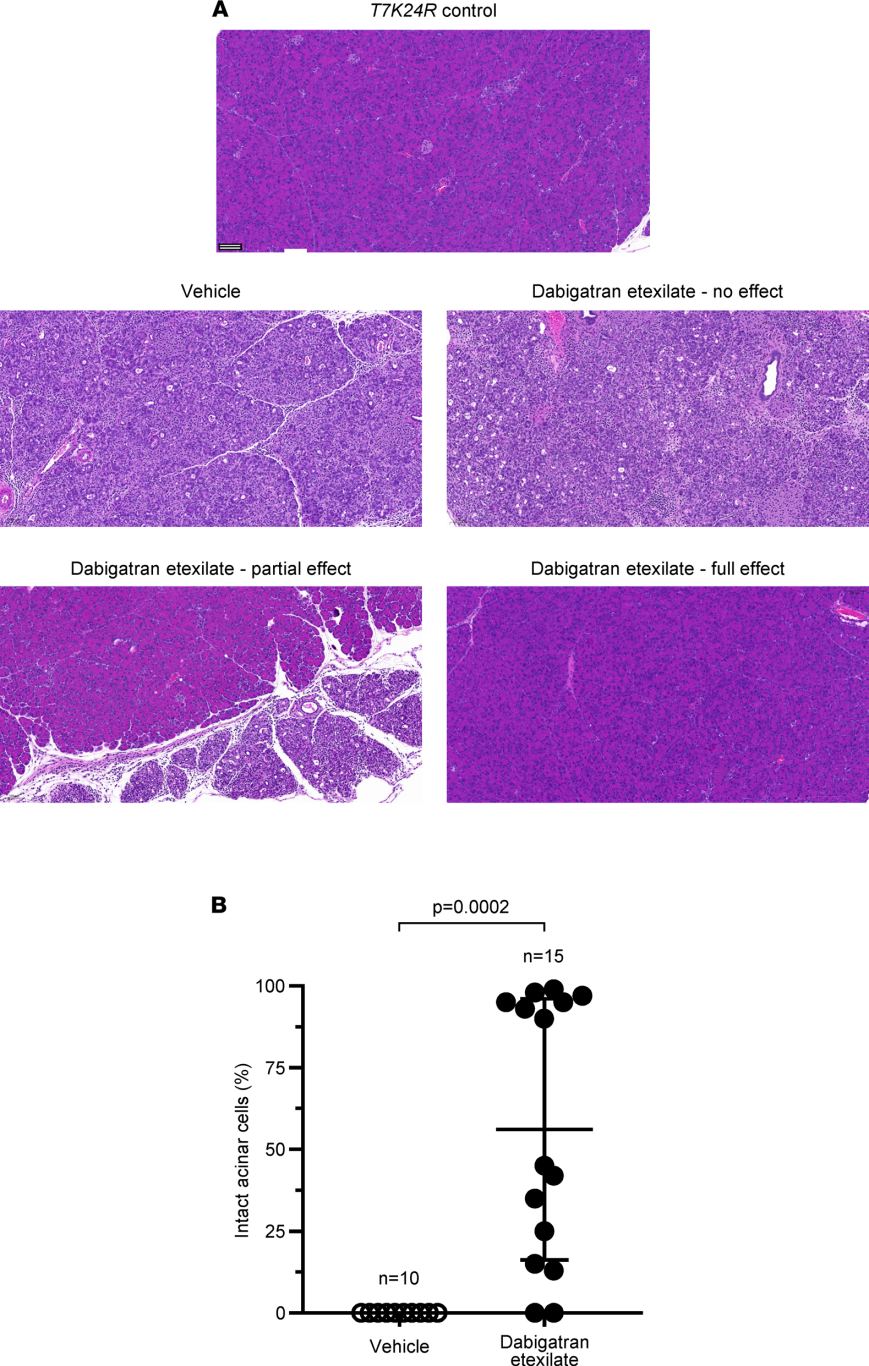
Effect of dabigatran etexilate on pancreas histology in cerulein-induced pancreatitis of *T7K24R* mice. Mice were given 8 hourly injections of cerulein, followed by a single oral gavage of 200 mg/kg dabigatran etexilate or vehicle administered 30 minutes after the last cerulein injection. Mice were euthanized 96 hours from the first cerulein injection. (**A**) Representative pancreas sections stained with hematoxylin-eosin from *T7K24R* mice given vehicle or dabigatran etexilate after the cerulein injections. For comparison, the normal pancreas histology of a control *T7K24R* mouse is shown. Scale bar corresponds to 100 μm. Higher magnification pictures of cerulein-induced pancreas pathology in *T7K24R* mice are shown in ref. 31. (**B**) Histological evaluation of acinar cell loss. Pancreas sections from mice given vehicle or dabigatran were visually scored for the presence of intact acinar cells. Individual data points with mean and standard deviation are shown. The difference of means between 2 groups was analyzed by 2-tailed unpaired *t* test.

Therefore, in our experiments, we induced pancreatitis in *T7K24R* mice by 8 hourly injections of cerulein and euthanized the mice 96 hours (i.e., 4 days) later. To test the effect of dabigatran etexilate, a single dose of the prodrug was administered 30 minutes after the last injection (Figure 5A). Negative control mice without pancreatitis and vehicle-treated positive control mice with pancreatitis served for comparison. When the body weight of mice at the beginning and at the end of the experiment was compared, vehicle-treated mice with pancreatitis showed a slight decrease (Figure 5B). This phenomenon is due to a transient digestive dysfunction associated with the rapid development of acinar atrophy (31). In contrast, dabigatran etexilate–treated *T7K24R* mice with pancreatitis showed no change in body weight by the end of the experiment, suggesting a protective effect of the drug. The pancreas weight of vehicle-treated *T7K24R* mice with pancreatitis was significantly reduced, to almost half the normal pancreas size (Figure 5C). The atrophic weight loss of the pancreas remained prominent even after the pancreas weight was normalized to body weight (Figure 5D). Remarkably, however, the pancreas weight of the dabigatran etexilate–treated mice with pancreatitis was significantly higher, in some cases approaching the values of control mice with no pancreatitis, suggesting that the drug prevented and/or reversed acinar atrophy to a large extent. *T7K24R* mice exhibited low plasma amylase activity 4 days after the induction of cerulein-induced pancreatitis, close to the levels seen in control mice without pancreatitis (Figure 5E). Interestingly, in a subset of dabigatran etexilate–treated mice with pancreatitis (4 of 15), we observed more than 3-fold higher plasma amylase activity values, suggesting ongoing acinar cell injury. Histological analysis of pancreata from 10 vehicle-treated and 15 dabigatran etexilate–treated mice with hematoxylin-eosin staining demonstrated widespread loss of intact acini in vehicle-treated mice (for higher magnification of pathological details, see ref. 31). A dramatic, complete protective effect of dabigatran etexilate was observed in almost 50% of the drug-treated mice (Figure 6A). A significant yet incomplete (30%–50% normal histology) effect was seen in about 20% of the mice, whereas in the remaining 30% of mice dabigatran showed limited efficacy, with less than 25% of normal acini preserved (Figure 6B). This group included 2 cases with no detectable effect. Notably, the 4 drug-treated mice with the elevated plasma amylase activity all had partial histological responses, with 13%, 15%, 35%, and 45% intact acini visible on pancreas sections. Overall, the proportion of intact acini in the dabigatran etexilate–treated group was significantly higher than in the vehicle-treated group, indicating that dabigatran is effective in this model of trypsin-dependent pancreatitis.

### Effect of orally gavaged dabigatran etexilate on spontaneous pancreatitis in T7D23A mice.

**Figure 7:**
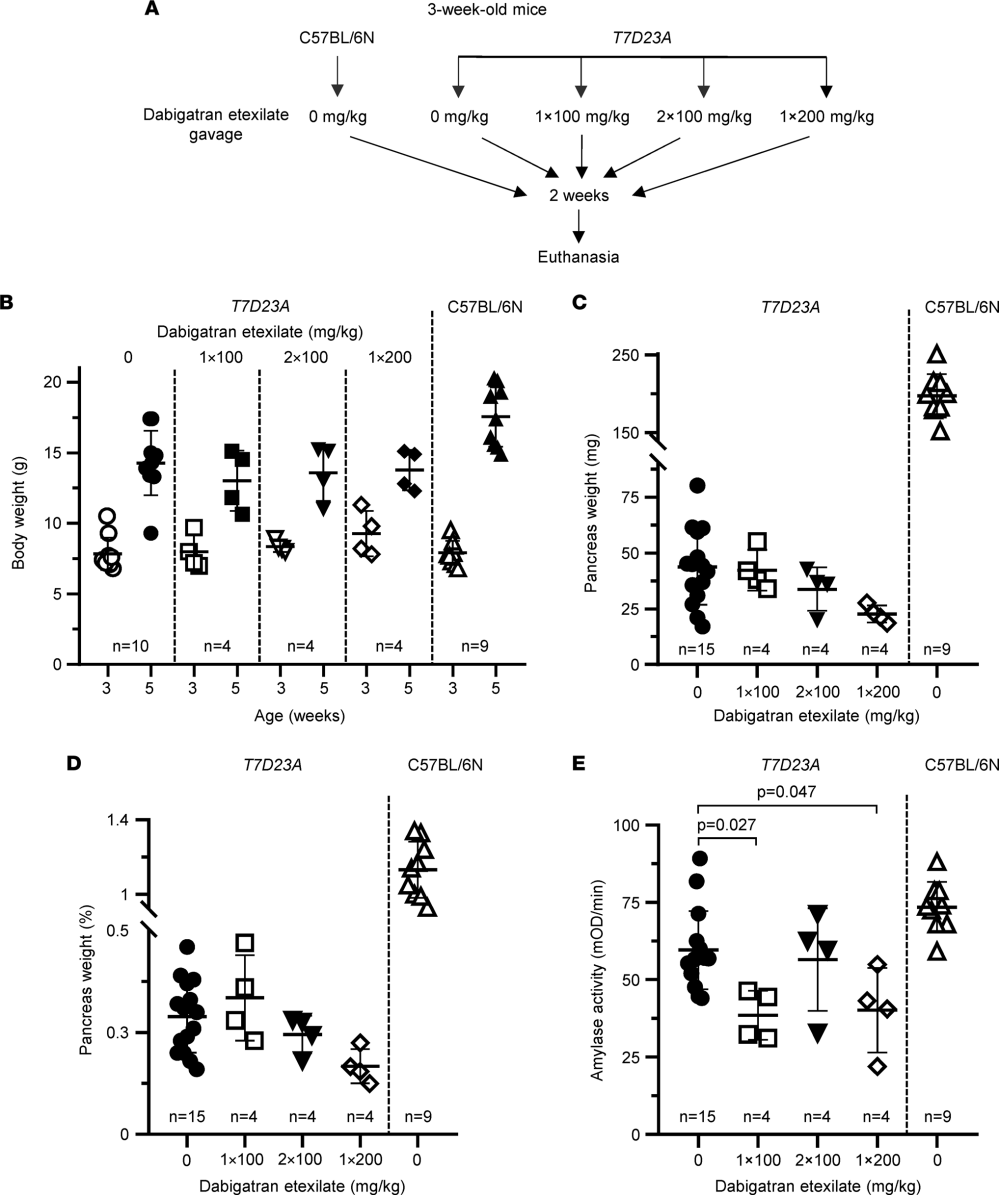
Effect of orally gavaged dabigatran etexilate on spontaneous pancreatitis in *T7D23A* mice. (**A**) Experimental design. Mice (3 weeks of age) were given 1 × 100 mg/kg, 2 × 100 mg/kg, or 1 × 200 mg/kg daily dose of dabigatran etexilate by intragastric gavage for 2 weeks. Untreated *T7D23A* and C57BL/6N mice served as controls. Mice were euthanized at 5 weeks of age. (**B**) Body weight of mice at the beginning (3 weeks) and end (5 weeks) of the experiment. (**C**) Pancreas weight in milligrams. (**D**) Pancreas weight as percentage of body weight. (**E**) Plasma amylase activity at 5 weeks of age (4 μL assayed). Individual data points with mean and standard deviation are shown. The difference of means between the groups was analyzed by 1-way ANOVA with Tukey-Kramer post hoc test.

**Figure 8:**
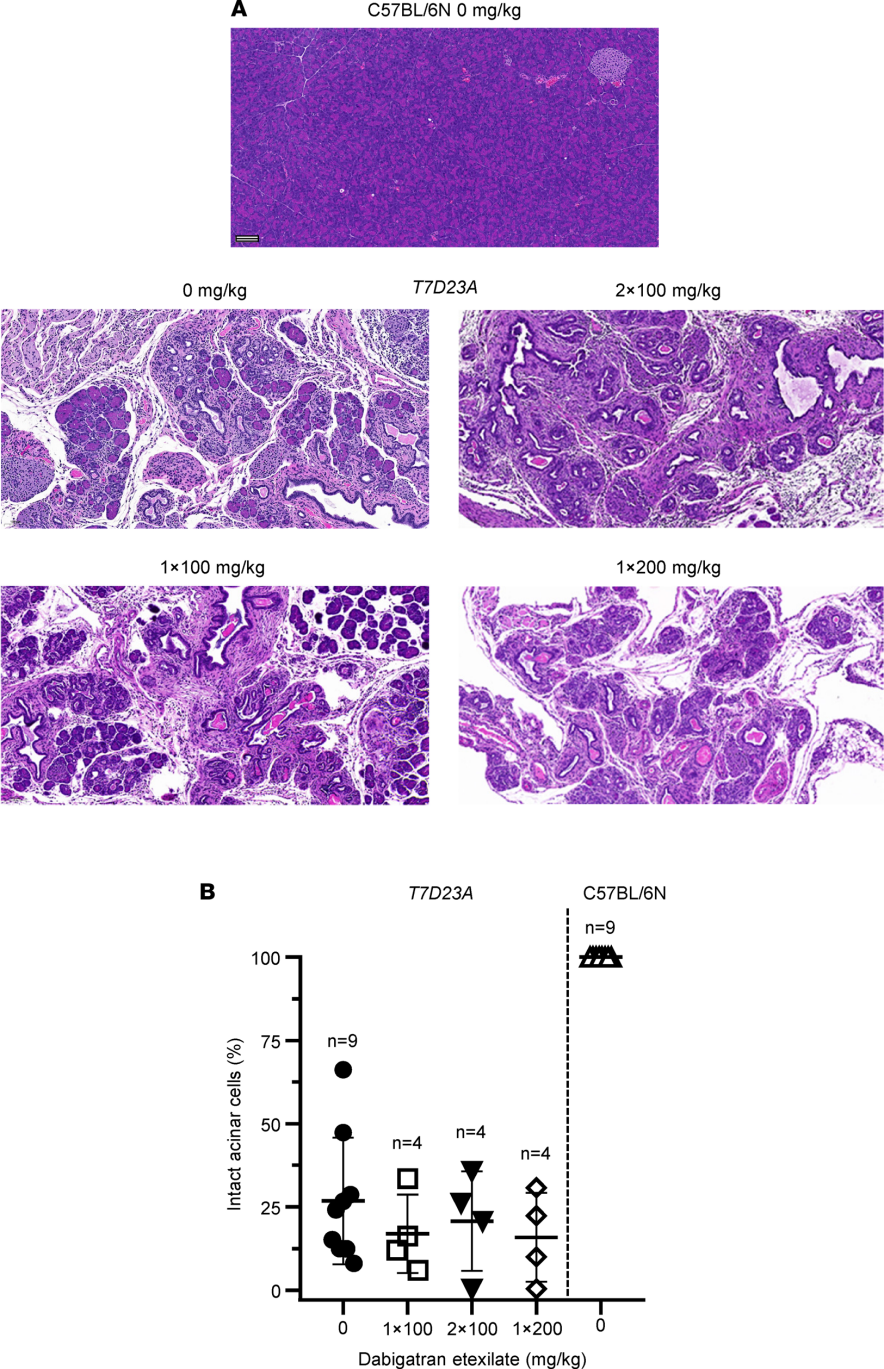
Effect of orally gavaged dabigatran etexilate on spontaneous pancreatitis in *T7D23A* mice. Mice (3 weeks of age) were given 1 × 100 mg/kg, 2 × 100 mg/kg, or 1 × 200 mg/kg daily dose of dabigatran etexilate by intragastric gavage for 2 weeks. Untreated *T7D23A* and C57BL/6N mice served as controls. Mice were euthanized at 5 weeks of age. (**A**) Representative pancreas sections stained with hematoxylin-eosin from untreated C57BL/6N and *T7D23A* control mice and *T7D23A* mice given dabigatran etexilate. Scale bar corresponds to 100 μm. Histological details of the spontaneous pancreatitis in *T7D23A* mice are also shown in ref. 29. (**B**) Histological evaluation of acinar cell loss. Pancreas sections were visually scored for the presence of intact acinar cells. Individual data points with mean and standard deviation are shown. The difference of means between the groups was analyzed by 1-way ANOVA with Tukey-Kramer post hoc test.

Next, we tested the effect of dabigatran etexilate in a more aggressive, spontaneous pancreatitis model. The *T7D23A* mouse strain carries the p.D23A mutation in mouse cationic trypsinogen (isoform T7), which is analogous to the p.D22G pancreatitis-associated human *PRSS1* mutation (29). The mutation increases autoactivation of trypsinogen about 50-fold and elicits spontaneous, early-onset (3–5 weeks of age), and progressive pancreatitis. In the first experiment, we treated 3-week-old *T7D23A* mice with various doses of dabigatran etexilate via intragastric gavage for 2 weeks (Figure 7A). The dosages used were once daily 100 mg/kg, twice daily 100 mg/kg, and once daily 200 mg/kg. As controls, untreated *T7D23A* and C57BL/6N mice were used. Mice were euthanized at 5 weeks of age. Based on prior experience, at the beginning of the experiment, the pancreas of 3-week-old *T7D23A* mice was either normal or may have had incipient AP (29). Conversely, by 5 weeks of age, all *T7D23A* mice were expected to have developed early CP. The body weight of mice was measured at the beginning (3 weeks) and at the end (5 weeks) of the experiment (Figure 7B). As expected, during this period, the mice gained weight, and this was unaffected by gavage treatment. The weight gain of *T7D23A* mice was slightly lower relative to the C57BL/6N parent strain. Compared with untreated C57BL/6N mice, the pancreas weight of untreated *T7D23A* mice was markedly lower, due to the massive pancreas atrophy associated with their early CP (Figure 7C) (29). This large difference persisted even after normalization of the pancreas weight to the body weight of the mice (Figure 7D). In stark contrast to the effect seen with *T7K24R* mice, dabigatran etexilate treatment did not improve the pancreas weight of *T7D23A* mice. Curiously, a clear trend of worsening atrophy emerged with increasing dabigatran dosages, even though the differences did not reach statistical significance. As expected, plasma amylase activity was reduced in untreated *T7D23A* mice relative to C57BL/6N mice, though the difference did not reach statistical significance (Figure 7E). In agreement with their smaller pancreas weights, drug-treated *T7D23A* mice had significantly lower amylase levels relative to the untreated *T7D23A* controls. Histological analysis of pancreata revealed comparable CP-like disease in all groups of *T7D23A* mice whereas C57BL/6N controls showed normal pancreas morphology (Figure 8A). For details of the histological phenotype of pancreatitis in *T7D23A* mice, the reader is referred to the original publication (29). Quantitative analysis of intact acini per visual field showed the expected dramatic cell loss in the pancreata of *T7D23A* mice, but no appreciable difference was seen between the untreated and treated groups (Figure 8B). Taken together, the results from this experiment indicated that dabigatran etexilate introduced by intragastric gavage did not ameliorate the spontaneous pancreatitis of *T7D23A* mice. Furthermore, under certain dosing protocols, the drug seemed to worsen the disease slightly.

### Effect of feeding with dabigatran etexilate–containing chow on spontaneous pancreatitis in T7D23A mice.

**Figure 9:**
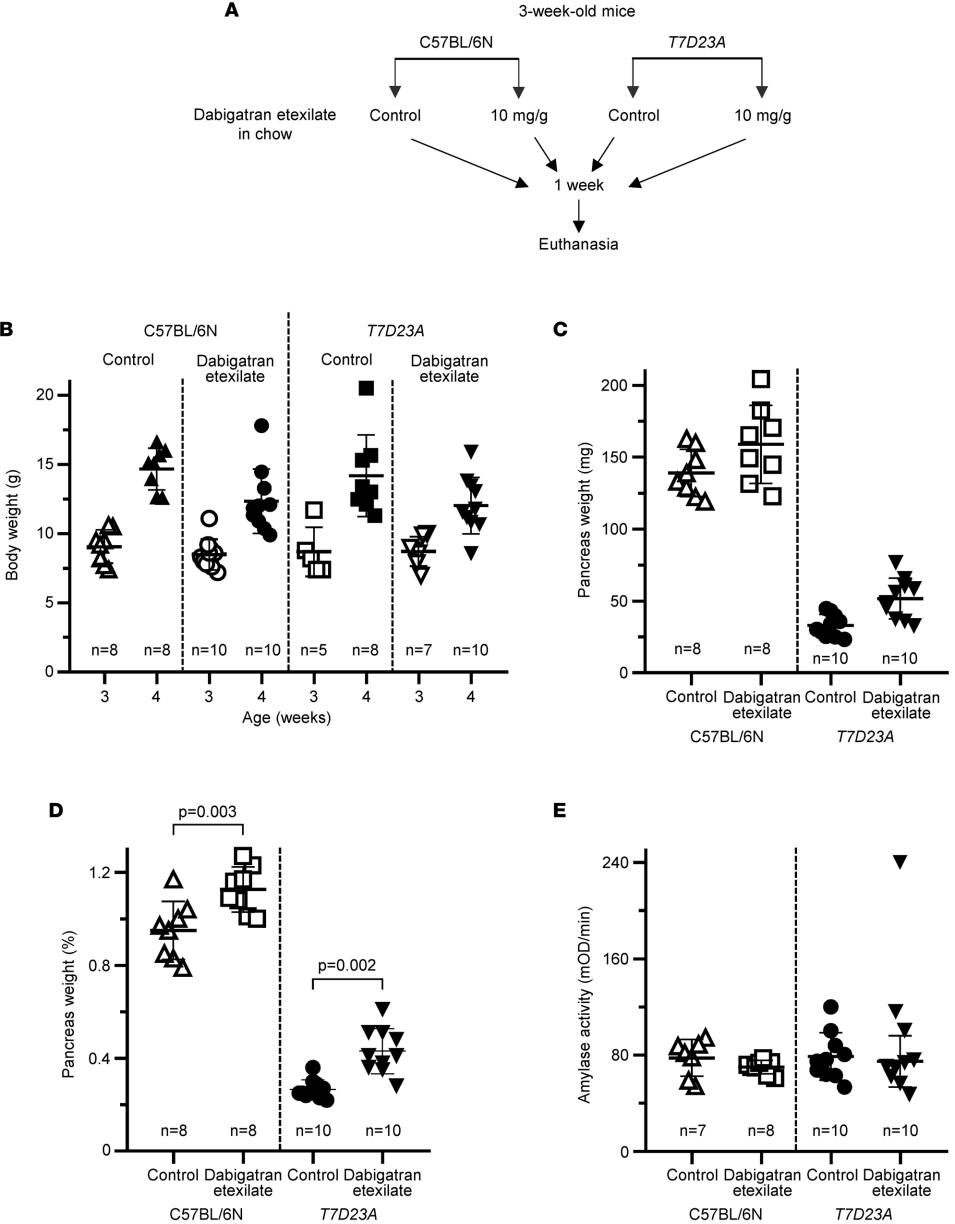
Effect of dabigatran etexilate feeding on spontaneous pancreatitis in *T7D23A* mice. (**A**) Experimental design. C57BL/6N and *T7D23A* mice were given solid chow with or without 10 mg/g dabigatran etexilate from 21 to 28 days of age. Mice were euthanized at 28 days of age. (**B**) Body weight at the beginning (3 weeks) and end (4 weeks) of the experiment. (**C**) Pancreas weight in milligrams. (**D**) Pancreas weight as percentage of body weight. (**E**) Plasma amylase activity (4 μL assayed). Individual data points with mean and standard deviation are shown. The difference of means between 2 groups was analyzed by 1-way ANOVA with Tukey-Kramer post hoc test. The outlier data point was excluded from the statistical calculations.

**Figure 10:**
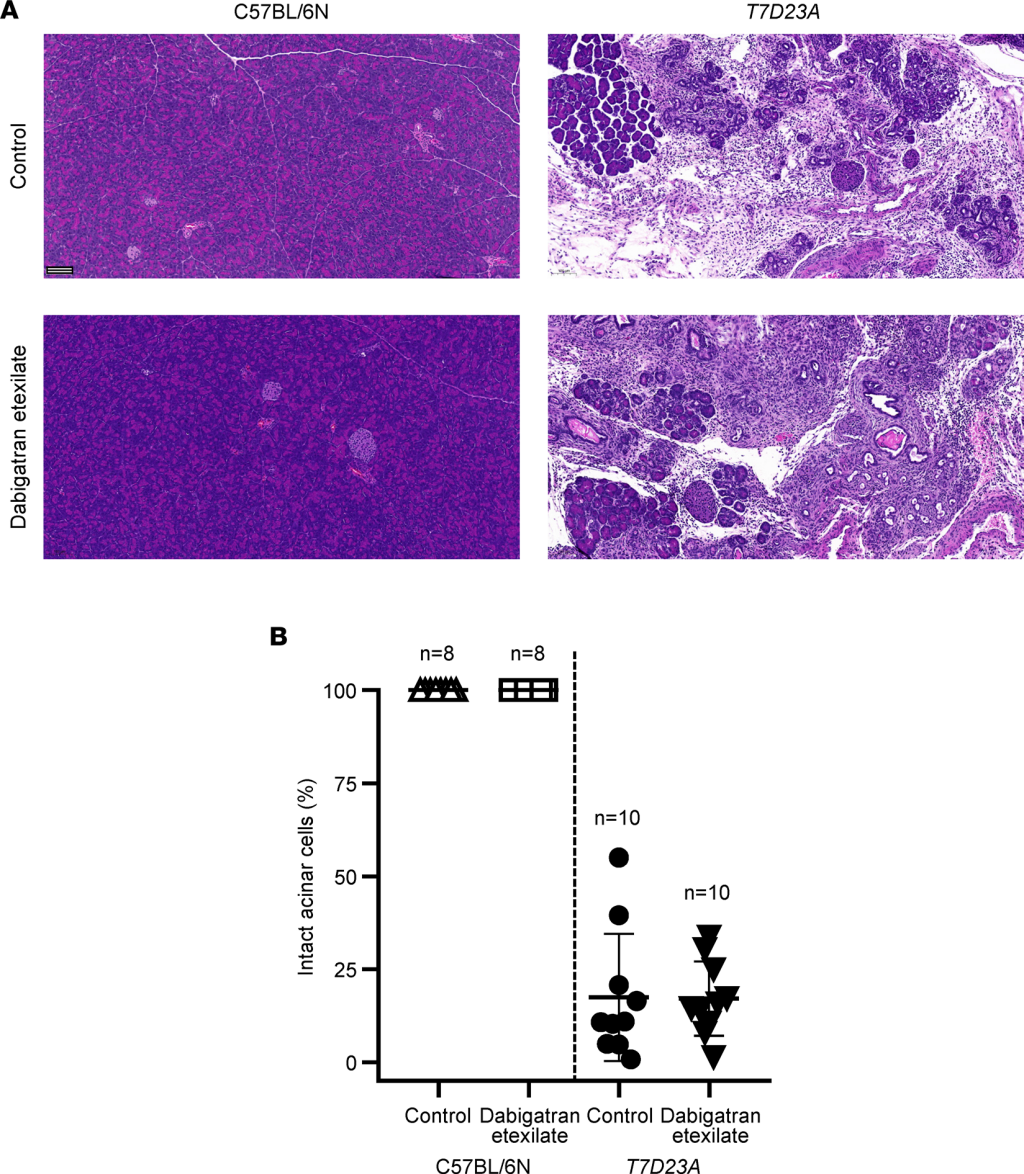
Effect of dabigatran etexilate feeding on spontaneous pancreatitis in *T7D23A* mice. C57BL/6N and *T7D23A* mice were given solid chow with or without 10 mg/g dabigatran etexilate from 21 to 28 days of age. Mice were euthanized at 28 days of age. (**A**) Representative pancreas sections stained with hematoxylin-eosin. Scale bar corresponds to 100 μm. Histological details of the spontaneous pancreatitis in *T7D23A* mice are also shown in ref. 29. (**B**) Histological evaluation of acinar cell loss. Pancreas sections were visually scored for the presence of intact acinar cells. Individual data points with mean and standard deviation are shown. The difference of means between the groups was analyzed by 1-way ANOVA with Tukey-Kramer post hoc test.

Based on the results of the gavage experiments described above, we speculated that the *T7D23A* mouse model may require sustained drug levels in the blood to achieve full inhibition of pancreatic trypsins and prevention/reversal of disease. Therefore, we tested whether feeding the mice with solid chow containing dabigatran etexilate would be efficacious. We fed 3 week-old-mice for 1 week and euthanized the mice at the age of 4 weeks (Figure 9A). There were 4 experimental groups, treated and untreated C57BL/6N controls, and treated and untreated *T7D23A* mice. Each group gained weight similarly during the 1-week treatment, indicating that mice readily consumed the dabigatran etexilate–containing chow (Figure 9B). When comparing the pancreas weight of treated and untreated mice, we observed a small increase in the drug-treated groups of both strains, indicating that this change is likely unrelated to a drug effect on pancreatitis (Figure 9C). The difference in pancreas weights reached statistical significance when normalized to body weight (Figure 9D). Long-term feeding of mice with trypsin inhibitors causes the pancreas weight to increase, due to luminal trypsin inhibition and a feedback mechanism that increases plasma cholecystokinin levels (44–48). Presumably, in our experiments, some of the unabsorbed dabigatran etexilate was converted to active dabigatran in the gut lumen and inhibited intestinal trypsins. Plasma amylase activities were comparable in all 4 groups of mice (Figure 9E). Finally, histological analysis showed normal pancreata in C57BL/6N mice and early CP in *T7D23A* mice (Figure 10A). Quantitative assessment of intact acini demonstrated no effect of dabigatran etexilate treatment on pancreatitis severity (Figure 10B). Taken together, the results indicate that feeding *T7D23A* mice with solid chow containing dabigatran etexilate did not prevent or improve their spontaneous pancreatitis.

## Discussion

In the present study, we tested the hypothesis that the recently reported therapeutic effect of dabigatran etexilate in experimental pancreatitis of transgenic PRSS1^R122H^ mice was due to the trypsin-inhibitory activity of dabigatran (34). To this end, first we modeled binding of dabigatran to trypsin by homology-based docking, then performed inhibition assays with dabigatran against a panel of human, mouse, and bovine trypsin paralogs, followed by preclinical studies with dabigatran etexilate on mouse models of trypsin-dependent pancreatitis. We found that dabigatran was readily docked into the substrate binding pocket of trypsin, and it potently inhibited all trypsin isoforms tested with similar efficacy. Compared with its parent compound, benzamidine, dabigatran showed several hundred-fold improved inhibitory activity. The inhibition was competitive, confirming that dabigatran blocks substrate binding to trypsin. Before the preclinical mouse studies, we confirmed that oral administration of dabigatran etexilate in mice can produce high enough dabigatran levels in the blood to achieve trypsin inhibition. A single gavage of dabigatran etexilate (100 mg/kg dose) resulted in peak dabigatran concentrations (~2.5 μg/mL) more than 100-fold higher than the *K_i_* values against trypsin isoforms. However, dabigatran levels fell rapidly under these conditions, and the inhibitory activity was almost completely lost within a few hours. When dabigatran etexilate was introduced to mice by chronic feeding of solid chow containing the prodrug (10 mg/g dose), relatively steady plasma concentrations were achieved (~300–400 ng/mL), which were about 10-fold higher than the *K_i_* values against trypsins. We add, as a caveat, that we do not know how well dabigatran penetrates the pancreas, which is the site of trypsin inhibition to prevent pancreatitis. We were unable to measure dabigatran levels in the pancreas due to interference of pancreatic proteases with the HEMOCLOT functional assay. It is important to note that 3 times daily administration of 100 or 200 mg dabigatran etexilate to human volunteers resulted in steady-state plasma levels of dabigatran of around 50 and 100 ng/mL, respectively (40). Since the common dosing of Pradaxa in clinical practice is two 150 mg capsules daily (39), the resultant dabigatran plasma concentrations are unlikely to exert significant trypsin-inhibitory activity in the pancreas.

We tested the effect of dabigatran etexilate on trypsin-dependent pancreatitis in 2 mouse models we recently developed. First, homozygous *T7K24R* mice were used (30), which are phenotypically similar to the PRSS1^R122H^ model (34) in that they develop progressive, CP-like pancreatitis after an acute episode of cerulein-induced pancreatitis (31). We observed high intrapancreatic trypsin activity in cerulein-treated *T7K24R* mice, suggesting that trypsin-inhibitory therapy would be beneficial. Our experiments supported the published efficacy of dabigatran etexilate against cerulein-induced progressive CP, with the following observations. We found that a single gavage of the prodrug given within an hour after the last cerulein injection was sufficient for therapeutic activity. Furthermore, we observed variability with respect to the therapeutic effect. We speculate that cerulein-induced pathological trypsin activity in the pancreas has to be inhibited within a short time frame before disease progression is inevitable. Efficient inhibition depends on high enough plasma concentrations of dabigatran, which, in turn, are determined by the technical success of the gavage and the rate of prodrug absorption. The 1-hour peak concentration of plasma dabigatran after a single gavage of the prodrug showed significant variability (see Figure 3A), supporting our notion that this may be the critical factor in therapeutic variability. Due to limitations in solubility and gavage volume, we were unable to test higher dabigatran etexilate doses in this experimental setting. Taken together, the observations verify the proposed therapeutic potential of dabigatran etexilate in trypsin-dependent pancreatitis. Finally, we note that it was not feasible to pretreat mice with dabigatran etexilate before induction of pancreatitis, because subsequent cerulein injections would cause fatal bleeding due to the anticoagulant activity of dabigatran.

Next, we used heterozygous *T7D23A* mice, which is a relatively aggressive, trypsin-dependent CP model with rapid, spontaneous disease development and severe end-stage disease (29). We tested the effect of dabigatran etexilate early in the disease course, utilizing 1–2 week treatment protocols. The prodrug was either introduced by daily gavages or mixed with solid chow. Unexpectedly, we found no therapeutic effect whatsoever with either method. Based on our observations with the *T7K24R* mice discussed above, we believe that dabigatran concentrations we achieved in *T7D23A* mice were insufficient to exert a meaningful trypsin-inhibitory effect in the pancreas. With once or twice daily gavage treatment, only the short-lived peak levels might have been sufficiently high, while feeding solid chow mixed with the prodrug resulted in relatively steady but much lower blood concentrations of dabigatran. We also note that *T7K24R* and *T7D23A* mice carry trypsinogen mutants that exhibit 5-fold and 50-fold increased autoactivation, respectively. Thus, due to the stronger biochemical and pathological phenotype, *T7D23A* mice may require higher dabigatran levels than *T7K24R* mice for successful treatment of pancreatitis.

We observed potentially problematic effects of dabigatran etexilate treatment in *T7D23A* mice that will require further investigation in the future. In the gavage experiments, there was a clear trend for the pancreas atrophy to show worse parameters relative to the untreated controls. A dose dependence was also apparent, with the largest effect seen with twice daily doses. Plasma amylase activities were also lower in the drug-treated groups, in agreement with the more pronounced pancreas atrophy. While an explanation to this observation is not readily apparent, we believe that the relatively rapid fluctuations in dabigatran levels may be responsible for the phenomenon. The transient trypsin inhibition in the pancreas may produce a rebound effect once dabigatran levels fall. This may also explain why plasma amylase levels of dabigatran etexilate–treated *T7K24R* mice with an incomplete response were elevated (see Figure 5E). In this case, a more localized rebound effect may have occurred, resulting in acinar cell damage, as judged by the amylase release.

Chronic feeding of *T7D23A* mice with dabigatran etexilate mixed in with chow resulted in small but measurable increases in their pancreas weight. The pancreas weight gain also occurred with C57BL/6N mice, indicating that this side effect was unrelated to pancreatitis treatment. The growth-stimulating effect of trypsin inhibitor feeding on the rodent pancreas has been extensively documented (44–48), and we believe this was the case here too. Some of the dabigatran etexilate likely was converted to dabigatran in the gut and inhibited luminal trypsins, which, in turn, increased plasma cholecystokinin levels that had a direct trophic effect on the pancreas. This feedback regulation may confound preclinical experiments testing orally administered trypsin inhibitors in rodents, particularly if pancreas size, weight, and function are used as treatment outcome measures. This concern is supported by a study showing that severity of experimental pancreatitis increased after rats and mice were fed camostat for 2 weeks (49). A similar feedback mechanism of functional adaptation seems to exist in humans; however, this may not be clinically relevant (50).

The premise of our studies was that dabigatran inhibits trypsin and thereby ameliorates pancreatitis. Although the results are consistent with this hypothesis, we cannot formally rule out other mechanistic pathways that may inhibit pancreatic inflammation and/or improve pancreas regeneration. In their seminal paper, the Ji laboratory speculated that the anticoagulant activity of dabigatran may be partly responsible for its therapeutic effectiveness in pancreatitis (34). They presented intriguing data showing that the factor Xa inhibitor apixaban (100 mg/kg), which is devoid of trypsin-inhibitory activity, had no therapeutic activity whatsoever. However, when combined with camostat (200 mg/kg), it became highly effective, while camostat alone showed only a partial effect. One possible interpretation of these results is that anticoagulation alone has no therapeutic effect. However, it may improve tissue penetration of the trypsin inhibitor camostat, thereby rendering it more effective. Previous studies demonstrated that heparin improves outcomes of cerulein-induced pancreatitis in rodents, presumably by eliminating fibrin deposits and improving circulation and oxygenation (51, 52). Furthermore, a recently published retrospective clinical cohort analysis found that systemic anticoagulation is associated with decreased mortality and morbidity in AP, suggesting at least some role for anticoagulation in pancreatitis therapy (53). Finally, thrombin inhibition by dabigatran has been shown to reduce proinflammatory macrophages in atherosclerotic lesions (54) and to improve organ fibrosis in various mouse models (55–57).

The goal of the present study was to offer proof of concept that pancreatic trypsin inhibition can have a therapeutic effect in pancreatitis. We chose to focus on dabigatran etexilate, because this drug is already in clinical use worldwide, is orally bioavailable, and, aside from the adverse effects related to anticoagulation, has an excellent safety profile. Despite the mixed results, we view this study as strongly encouraging because the observations demonstrated that benzamidine derivatives can be highly effective trypsin inhibitors with demonstrable therapeutic efficacy. We note that future clinical trials of trypsin inhibitors in pancreatitis should focus on *PRSS1*-related hereditary pancreatitis and other trypsin-mediated forms of the disease. Furthermore, trial designs should consider that trypsin inhibition is expected to prevent or dampen disease initiation but not severity outcomes, such as organ failure, caused by lipotoxicity (58). Finally, the results warrant revisiting the potential therapeutic utility of other compounds with trypsin-inhibitory activity, such as gabexate, camostat, and nafamostat, in trypsin-dependent pancreatitis.

## Methods

### Materials.

Bovine trypsin (catalog number LS003707) was purchased from Worthington Biochemical Corporation and was active-site titrated with *p*-nitrophenyl *p’*-guanidino-benzoate (catalog number N-8010, MilliporeSigma) (59). Ecotin was produced and purified as reported previously (60). The concentration of ecotin was determined by titration against active-site titrated bovine trypsin. Recombinant human pro-enterokinase (catalog number 1585-SE-010) was purchased from R&D Systems and was activated with human cationic trypsin. The trypsin substrate GPR-pNA (catalog number 4000768.0100) was purchased from Bachem Americas. Benzamidine hydrochloride (code number 401790050) was obtained from Acros Organics (through Thermo Fisher Scientific) and dissolved in distilled water to prepare a 100 mM stock solution, which was further diluted with distilled water to a 10 mM working solution. Dabigatran etexilate mesylate (catalog number D100150) and dabigatran (catalog number D100090) were purchased from Toronto Research Chemicals. Dabigatran used for biochemical studies was dissolved in 0.1N HCl first to prepare an 800 μM stock solution, which was further diluted with distilled water to a 10 μM working solution.

### Modeling dabigatran binding to trypsin.

The dabigatran chemical structure was downloaded from the PubChem database (https://pubchem.ncbi.nlm.nih.gov/) as canonical SMILES string. The human mesotrypsin structure 1H4W was downloaded from PDB (42). The docking was carried out by the docking server ClusPro LigTBM (https://ligtbm.cluspro.org/) (61). The program performed a similarity search in the PDB database to find all trypsin structures or homologs cocrystallized with ligands similar to dabigatran as templates. An ensemble of 1,000 initial conformations was generated for the ligand. For each template, all conformers were aligned to the template, and only 1 conformer with the lowest root mean square deviation was retained. The resulting protein-ligand structures were subjected to restrained all-atom energy minimization using a CHARMM-based energy function (62) with a GBSA-type solvation term (Analytical Continuum Electrostatics) to remove possible clashes and “relax” the ligand.

### Expression, purification, and activation of trypsinogens.

Recombinant human and mouse trypsinogens were expressed in *Escherichia coli* BL21(DE3) (Invitrogen), refolded in vitro, and purified by ecotin affinity chromatography (Pharmacia FPLC), according to published protocols (60, 63, 64). Sulfated human trypsinogens were purified from archived samples of human pancreatic juice, as described previously, with minor modifications (65, 66). Briefly, 50 mg freeze-dried pancreatic juice was dissolved in 2 mL 10 mM HCl, clarified by centrifugation (21,000*g*, 5 minutes, 4°C), and loaded onto a MonoQ 5/50 GL column equilibrated with 20 mM Tris-HCl (pH 8.0). Proteins were eluted with a 0–0.5 M NaCl gradient at 1 mL/min flow rate for 30 minutes. The fractions containing the cationic and anionic trypsinogens were further purified with ecotin affinity chromatography. The concentration of trypsinogen preparations was estimated from their UV absorbance at 280 nM using the extinction coefficients reported previously for mouse trypsinogens (64) and 37,525, 38,890, and 41,535/M/cm for human cationic trypsinogen (PRSS1), anionic trypsinogen (PRSS2), and mesotrypsinogen (PRSS3), respectively. To activate trypsinogen to trypsin, approximately 1 μM solution was incubated at 37°C with 28.2 ng/mL human enterokinase (final concentration) in 0.1 M Tris-HCl (pH 8.0), 10 mM CaCl_2_, and 0.05% Tween 20 for 1 hour. The activation reaction was followed by measuring trypsin activity: 2 μL trypsin was diluted in 48 μL assay buffer [0.1 M Tris-HCl (pH 8.0), 1 mM CaCl_2_, and 0.05% Tween 20], and 150 μL of 200 μM GPR-pNA trypsin substrate (in assay buffer) was added. Substrate cleavage resulting in the release of the yellow *p*-nitroaniline was monitored for 1 minute in a Spectramax Plus 384 microplate reader at 405 nm.

### Active-site titration of trypsin.

The concentration of trypsin solutions was determined by titration with the trypsin inhibitor ecotin. Briefly, a 2-fold serial dilution of ecotin was prepared in 100 μL assay buffer in a microplate, 100 μL of trypsin solution was added, and the mixture was incubated at 24°C for 30 minutes. The final ecotin concentrations in the 200 μL volume were 0, 0.78, 1.56, 3.1, 6.25, 12.5, 25, and 50 nM, and the final nominal trypsin concentration was 10 nM. Trypsin activity was measured after adding 5 μL of 6 mM GPR-pNA substrate to each well, as described above. The trypsin activity was plotted as a function of the ecotin concentration, and the true trypsin concentration was determined from the extrapolated *x* intercept of the linear portion of the inhibition curve.

### Enzyme kinetic measurements.

Michaelis-Menten kinetic parameters of trypsin isoforms were determined with the chromogenic substrate GPR-pNA at 24°C. The trypsin concentration was 1 nM and the substrate concentration was varied typically between 1.56 and 200 μM in a final volume of 200 μL assay buffer. The substrate concentration range was 3.1–400 μM for mouse trypsin isoform T7 and 0.4–50 μM for mouse trypsin isoform T20. *K_m_* and *k_cat_* values were calculated from hyperbolic fits to plots of reaction velocity versus substrate concentration.

### Measuring competitive inhibition by benzamidine and dabigatran.

Trypsin isoforms were preincubated with increasing concentrations of benzamidine or dabigatran for 15 minutes at 24°C, in 100 μL assay buffer. Michaelis-Menten kinetic parameters were then measured after adding 100 μL trypsin substrate, as described above. The final trypsin concentration in the assay was 1 nM, the final benzamidine concentrations were from 12.5 to 100 μM, and the final dabigatran concentrations were from 25 to 200 nM, as indicated in Tables 1 and 2. To determine the *K_i_*, the *K_m_* values were plotted as a function of the inhibitor concentration, and the *K_i_* was calculated by dividing the *y* axis intercept with the slope of the linear fit. This value corresponds to the negative of the *x* axis intercept. Alternatively, the substrate saturation curves obtained in the absence and presence of the increasing inhibitor concentrations were globally fitted to the competitive inhibition equation y = v_max_ × (x/[*K*_mObs_+ x]), where y is the reaction velocity, v_max_ is the maximal velocity, x is the substrate concentration, and *K*_mObs_ = *K_m_* × (1 + [I]/*K_i_*), where [I] is the inhibitor concentration. Experiments were performed 3 times and each experiment was analyzed separately. The reported *K_i_* values thus represent the mean (± standard deviation) of 3 determinations. Kinetic analysis was performed with the Prism 8 program (GraphPad).

### Experimental animals.

The generation and properties of the *T7D23A* and *T7K24R* mice carrying trypsinogen mutations were described recently (29, 30). Heterozygous *T7D23A* and homozygous *T7K24R* mice were used. Protocols for genotyping have been reported earlier (29, 30). C57BL/6N mice were purchased from Charles River Laboratories or produced in our breeding facility from the same stock. Both male and female mice were studied. The number of mice used in the experiments is shown in the figures.

### Cerulein-induced pancreatitis in T7K24R mice.

Pancreatitis was induced in *T7K24R* mice with 8 hourly intraperitoneal injections of the secretagogue peptide cerulein used in a dose of 50 μg/kg. Cerulein (catalog number C9026, MilliporeSigma) was dissolved in sterile normal saline at 10 μg/mL concentration. Mice were sacrificed 1, 24, 48, 72, or 96 hours from the first cerulein injection, as indicated, and the pancreas and blood were harvested. Details of histological analysis and measurement of plasma amylase (4 μL assayed) were reported previously (29–31).

### Intrapancreatic protease activity in T7K24R mice.

Trypsin and chymotrypsin activities were measured from freshly prepared pancreas extracts of C57BL/6N and *T7K24R* mice using our recently published protocol (67). Activity was expressed as the rate of substrate cleavage in relative fluorescent units per second, normalized to the total protein concentration in milligrams.

### Dabigatran etexilate treatment of T7D23A and T7K24R mice.

Mice were administered dabigatran etexilate orally either by intragastric gavage of a 20 mg/mL solution to a final dose of 100 or 200 mg/kg using a 24 gauge, 1 inch long feeding needle (catalog number FN7900, Roboz Surgical) or by feeding with solid chow containing the prodrug in 10 mg/g concentration, as indicated in the experimental design. With these treatment protocols, no bleeding, morbidity, or mortality were observed. We note, however, that after administration of dabigatran etexilate, further injections or biopsies were not possible without the risk of significant bleeding. We also inspected hematoxylin-eosin–stained sections of liver, intestine, and kidney from mice treated with dabigatran etexilate or vehicle but found no signs of organ damage. Control mice were given gavage of the vehicle solution (Captisol; see below) or regular chow.

### Preparation of gavage solution and solid chow containing dabigatran etexilate.

Dabigatran etexilate solution was always freshly prepared before use by dispersing 20 mg prodrug in 1 mL of 35% Captisol solution in a bath-type sonicator (5510 DTH Ultrasonic Cleaner, Branson) for 5–10 minutes at 24°C. Captisol (β-cyclodextrin sulfobutyl ethers, sodium salts, lot number NC-04A-180185) was obtained from Cydex Pharmaceuticals (through Thermo Fisher Scientific) and was dissolved in sterile normal saline (3.5 g in 10 mL). Dabigatran etexilate–containing chow was prepared using regular chow (5053 PicoLab Rodent Diet 20, LabDiet), which was pulverized with a pestle in a ceramic mortar. Solid dabigatran etexilate mesylate (1 g) was mixed with 100 g of powdered chow (10 mg/g final prodrug concentration), and sterile distilled water was added until the chow became malleable. The mixture was then compressed into pellets using the barrel of a 10 mL plastic syringe and a plunger. The prodrug-containing pellets were allowed to dry at 24°C for 2 days and stored at 4°C until use.

### Estimation of absorbed dabigatran etexilate.

The oral bioavailability of dabigatran etexilate in humans is around 6.5%, and it is independent of dose and not influenced by food (40). Accordingly, a 100 mg/kg dose of intragastric gavage can provide a 0.13 mg single dose of systemically absorbed dabigatran etexilate in a 20 g mouse. When dabigatran etexilate is given mixed with solid chow (10 mg prodrug/g chow), mice can consume approximately 4 g chow over a 24-hour period. This would yield a 2.6 mg daily dose of absorbed dabigatran etexilate.

### Determination of dabigatran plasma concentration.

Dabigatran in the blood plasma was quantified using the HEMOCLOT Thrombin Inhibitors (3 × 2.5 mL) kit (catalog number CK002L-RUO, Hyphen BioMed, purchased from Aniara Diagnostica) (68, 69). Reagents were diluted according to the manufacturer’s instructions. The BIOPHEN Dabigatran Plasma Calibrator set (catalog number 2222801-RUO, Hyphen BioMed, purchased from Aniara Diagnostica) was used to establish a calibration curve for the clotting time as a function of plasma dabigatran concentration. Mouse blood was collected immediately after CO_2_ euthanasia. To achieve rapid anticoagulation, 20 μL of a 3.2% solution of sodium citrate was pipetted into the conus of a needle before attaching it to a 1 mL syringe and collecting approximately 180 μL blood by cardiac puncture. Cellular blood elements were sedimented by centrifugation (2,000*g*, 15 minutes, 4°C); the plasma was saved and further diluted with normal saline before use (20 μL plasma was mixed with 160 μL saline). A clean microscope slide was placed on the surface of a block heater set at 37°C. Aliquots of reagents R1 and R2 (110 μL), and the calibrators or plasma specimen (60 μL), were pipetted onto the glass surface and preheated for 2 minutes. Reagent R1 (100 μL) was then mixed with 50 μL of the calibrator or plasma on the slide and incubated for 1 minute before addition of reagent R2 (100 μL). Time to the appearance of the first fibrin clot was measured. Clotting was detected by manual probing of the incubation mixture for fibrin threads with a small pipette tip.

### Statistics.

Results are given as mean values ± standard deviation or shown as individual data points with the mean ± standard deviation indicated. Differences between means were analyzed by unpaired 2-tailed *t* test for 2 groups and by 1-way ANOVA for multiple groups, with Tukey-Kramer post hoc analysis for pairwise comparison using Prism 8 (GraphPad). Statistical significance was defined as *P* < 0.05.

### Study approval.

Animal experiments were performed at the University of California Los Angeles (UCLA) with the approval and oversight of the Animal Research Committee, including protocol review and postapproval monitoring. The animal care program at UCLA is managed in full compliance with the US Animal Welfare Act, the US Department of Agriculture Animal Welfare Regulations, the US Public Health Service Policy on Humane Care and Use of Laboratory Animals, and the *Guide for the Care and Use of Laboratory Animals* (National Academies Press, 2011). UCLA has an approved Animal Welfare Assurance statement (A3196-01) on file with the US Public Health Service, NIH, Office of Laboratory Animal Welfare. UCLA is accredited by the Association for Assessment and Accreditation of Laboratory Animal Care International.

## Author contributions

MST conceived and directed the study. MST, ZGP, ZJ, AD, and SV designed the experiments. ZGP, ZJ, AD, BCN, and SV performed the experiments. All authors analyzed the data. MST wrote the manuscript; ZGP, ZJ, AD, and SV prepared the figures. All authors read and approved the manuscript.

## Figures and Tables

**Table 3:**
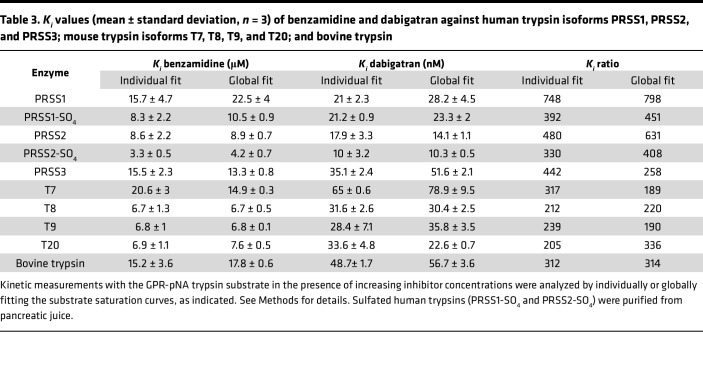
*K_i_* values (mean ± standard deviation, *n* = 3) of benzamidine and dabigatran against human trypsin isoforms PRSS1, PRSS2, and PRSS3; mouse trypsin isoforms T7, T8, T9, and T20; and bovine trypsin

**Table 2:**
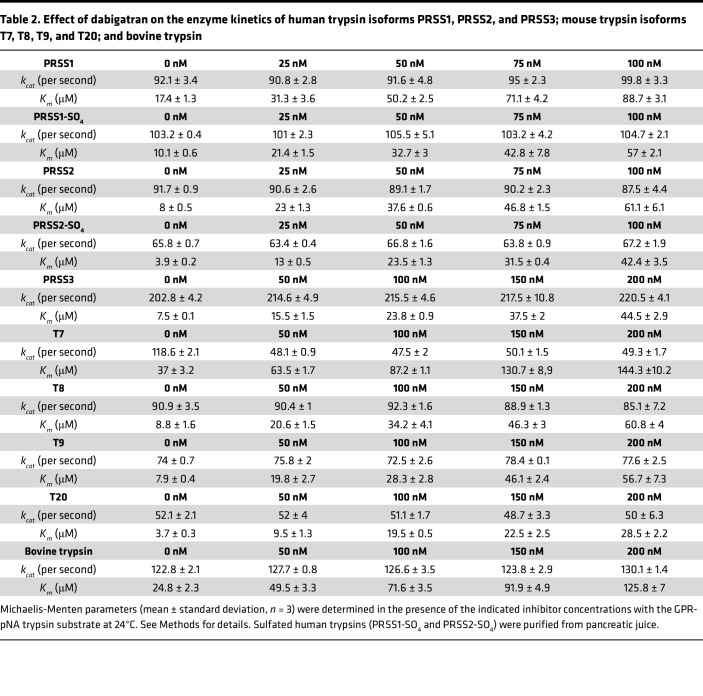
Effect of dabigatran on the enzyme kinetics of human trypsin isoforms PRSS1, PRSS2, and PRSS3; mouse trypsin isoforms T7, T8, T9, and T20; and bovine trypsin

**Table 1:**
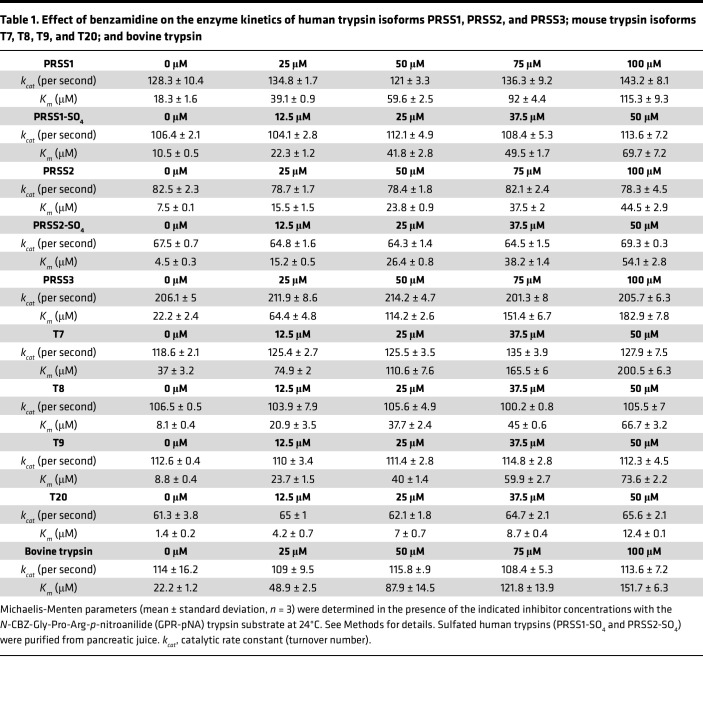
Effect of benzamidine on the enzyme kinetics of human trypsin isoforms PRSS1, PRSS2, and PRSS3; mouse trypsin isoforms T7, T8, T9, and T20; and bovine trypsin

## Version 1 09/22/2022

In-Press Preview

## Version 2 11/08/2022

Electronic publication
